# Cancer/Testis Antigens: “Smart” Biomarkers for Diagnosis and Prognosis of Prostate and Other Cancers

**DOI:** 10.3390/ijms18040740

**Published:** 2017-03-31

**Authors:** Prakash Kulkarni, Vladimir N. Uversky

**Affiliations:** 1 Institute for Bioscience and Biotechnology Research, University of Maryland, Rockville, MD 20850, USA; 2 Department of Molecular Medicine, Morsani College of Medicine, University of South Florida, Tampa, FL 33612, USA; 3 Laboratory of New methods in Biology, Institute for Biological Instrumentation, Russian Academy of Sciences, Pushchino 142290, Moscow Region, Russia

**Keywords:** biomarkers, prostate cancer, cancer/testis antigens, intrinsically disordered protein, prostate-associated gene 4 (PAGE4), nucleolar protein 4 (NOL4), centrosomal protein of 55 kDa (CEP55)

## Abstract

A clinical dilemma in the management of prostate cancer (PCa) is to distinguish men with aggressive disease who need definitive treatment from men who may not require immediate intervention. Accurate prediction of disease behavior is critical because radical treatment is associated with high morbidity. Here, we highlight the cancer/testis antigens (CTAs) as potential PCa biomarkers. The CTAs are a group of proteins that are typically restricted to the testis in the normal adult but are aberrantly expressed in several types of cancers. Interestingly, >90% of CTAs are predicted to belong to the realm of intrinsically disordered proteins (IDPs), which do not have unique structures and exist as highly dynamic conformational ensembles, but are known to play important roles in several biological processes. Using prostate-associated gene 4 (PAGE4) as an example of a disordered CTA, we highlight how IDP conformational dynamics may regulate phenotypic heterogeneity in PCa cells, and how it may be exploited both as a potential biomarker as well as a promising therapeutic target in PCa. We also discuss how in addition to intrinsic disorder and post-translational modifications, structural and functional variability induced in the CTAs by alternate splicing represents an important feature that might have different roles in different cancers. Although it is clear that significant additional work needs to be done in the outlined direction, this novel concept emphasizing (multi)functionality as an important trait in selecting a biomarker underscoring the theranostic potential of CTAs that is latent in their structure (or, more appropriately, the lack thereof), and casts them as next generation or “smart” biomarker candidates.

## 1. Introduction

Prostate cancer (PCa) is one of the most prevalent forms of cancer in older men over the age of 50. Worldwide, >1 million men are diagnosed with PCa each year and more than 300,000 die of the disease. Current US statistics show that one in five or six men will be diagnosed with PCa during their lifetime. In fact, by extrapolating statistical data from the past 40 years (1973–2013) it is estimated that in little over 100 years, one in two men will develop the disease [1]. Although these numbers appear daunting, only a fraction of those diagnosed have forms of the disease that can be considered to be “lethal” in nature.

As is true for other types of cancer, early diagnosis is believed to be crucial for the selection of the most successful and suitable PCa treatment strategy. Therefore, it follows that regular screening of men over the age 50 may be a logical thing to do. However, even today, there is no reliable test other than prostate-specific antigen (PSA), and there is no unanimous opinion in the medical community regarding the benefits of PSA screening [2,3]. Those who advocate regular screening believe that early diagnosis and treatment of PCa offers men a better chance to address the disease. On the other hand, urologists who recommend against regular screening note that because PCa is typically slow growing, the side effects of treatment would likely outweigh any benefit that might be derived from detecting the disease at a stage when it is unlikely to cause problems. Consistently, in 2012, the United States Preventive Services Task Force (USPSTF), an independent panel of experts in primary care and prevention that systematically reviews the evidence of effectiveness and develops recommendations for clinical preventive services, recommended against PCa screening in adult men of all ages. Indeed, in a roundtable discussion organized by Lee et al. to analyze studies of screening in two large randomized trials, it became obvious that the benefits of screening may not occur for 10 or more years after screening given the long natural history of the disease and that, perhaps as many as 1000 men will need to be screened and about 50 will need to be treated to save one life from PCa [4].

Many factors, including an increase in the aging population and widespread screening for PSA have contributed to the rise in the diagnoses of men who present early-stage (low or intermediate Gleason scores (GS)) or “low-risk” disease. While immediate treatment is recommended for patients with high GS (≥8), the appropriate treatment for patients with low GS (≤6) or intermediate GS (=7) remains ambiguous. Patients with low-risk disease are typically recommended the “wait and watch” or “active surveillance” protocol but are routinely monitored including repeat biopsies with the intention of avoiding treatment unless there is evidence of disease progression [5,6,7]. It is, therefore, not surprising that a staggering number of biopsies—>1 million in the US alone—are performed every year adding to the burgeoning healthcare cost and undesirable risk of serious complications requiring hospitalization [8].

While the intent of the active surveillance protocol is to minimize over-treatment, the concern is that active surveillance may miss the opportunity for early intervention of tumors that are seemingly low risk but that are actually aggressive. Indeed, despite the cautious approach, up to 40% of patients enrolled in active surveillance develop full-blown PCa [6]. Thus, a clinical dilemma today in the management of PCa is to distinguish men with aggressive disease who need definitive treatment from men whose disease does not require such intervention. Furthermore, accurate prediction of disease behavior is critical because radical treatment is associated with high morbidity.

Currently, at the time of diagnosis, most PCa cases present as localized disease and are preferentially treated by radical prostatectomy or radiation therapy with curative intent. During the last decade, a significant shift towards localized, well-differentiated tumors at radical prostatectomy (so-called stage migration) has occurred [9,10] perhaps due to the widespread use of PSA screening or a change in PCa biology [11], although, the latter would seem less likely. Therefore nowadays, PCa detected by PSA alone is often characterized by small-size and low-grade tumors in relatively younger male populations. In fact, it is reported that around 30% of cancers treated with radical prostatectomy in the US are “insignificant” tumors [12]. On the other hand, nearly 30% patients are reported to experience an isolated increase in serum PSA with long-term follow-up [13,14,15,16,17,18,19]. Therefore, it is important for physicians and patients to know the likelihood of disease progression following radical prostatectomy. Considered together, it follows that there is a critical need to identify reliable biomarkers that may be used for better diagnosis as well as to distinguish most of the low-GS tumors that will remain indolent from the few that are truly aggressive to better treat and manage PCa.

The advent of advanced technologies and sophisticated bioinformatics algorithms has fueled the discovery of novel biomarkers that include serum-, urine- and tissue-based assays that may supplement PSA testing, or even replace it over time (reviewed in [20,21,22,23,24,25,26,27,28]). These include extracellular vesicles [29], long noncoding RNAs [30,31], microRNAs [32,33,34] and circulating tumor cells (CTCs) [35,36], among many others. These advances have provided new insights into the individual patient’s tumor biology, and several biomarkers with specific indications for disease diagnosis, prediction and prognosis, as well as risk stratification of aggressive PCa at the time of diagnosis are now commercially available.

For example, Decipher™ (GenomeDx Biosciences, Vancouver, Canada) is a tool based on 22 genes that evaluates the risk of adverse outcomes (metastasis) after radical prostatectomy [37], while Oncotype DX^®^ (Genomics Health, Redwood City, CA, USA) was developed for use with fixed paraffin-embedded (FFPE) diagnostic prostate needle biopsies and measures expression of 12 cancer-related genes representing four biological pathways and five reference genes to calculate the Genomic Prostate Score (GPS) [38]. This assay has been analytically and subsequently clinically validated as a predictor of aggressive disease [38]. Prolaris (Myriad Genetics, Salth Lake City, UT, USA), on the other hand, is a 46-gene prognostic test that quantitatively determines the risk of recurrence in patients who have undergone prostatectomy. The assay measures the expression of 31 cell cycle progression (CCP) genes and 15 housekeeping genes that act as internal controls and normalization standards in each patient sample. The assay is also performed on FFPE samples and the results are reported as a numerical score along with accompanying interpretive information [39]. Of note, since the expression of CCP genes is likely to represent a fundamental aspect of tumor biology, the rationale for selecting these genes for prediction of outcome in PCa is based on a common biological function of the individual genes in this panel. Interestingly, the other two genomics tests also include genes associated with cell proliferation. Finally, ProMark^®^ (Metamark Corp., Waltham, MA, USA) s based on a multiplexed proteomics assay [40] and predicts PCa aggressiveness in patients found with similar features to Oncotype DX^®^. These biomarkers can be helpful for post-biopsy decision-making in low-risk patients and post-radical prostatectomy in selected risk groups. These biomarkers that are intended to be used in combination with the accepted clinical criteria (i.e., GS, PSA, clinical stage) to stratify PCa according to biological aggressiveness and direct initial patient management have gained considerable popularity; however, additional studies are needed to investigate the clinical benefit of these new technologies, the financial ramifications and how they should be utilized in clinics.

## 2. Cancer/Testis Antigens (CTAs) as Novel PCa Biomarkers

The cancer/testis antigens (CTAs) are a group of proteins that are typically restricted to the testis in the normal adult but are aberrantly expressed in several types of cancers [41]. To date, ~250 genes encoding CTAs have been identified [42] that can be broadly divided into two groups: CT-X antigens located on the X chromosome and non-X CTAs located on various autosomes. Furthermore, members of the CT-X antigens, in particular, are typically associated with advanced disease characterized by poorer outcomes in several types of cancers, including PCa [43,44,45,46,47,48]. Because of these intriguing expression patterns, the CTAs serve as unique biomarkers for cancer diagnosis/prognosis.

A systematic study by Suyama et al. [48] using a custom DNA microarray revealed that several CT-X antigens from melanoma-associated antigen A/chondrosarcoma-associate gene (MAGE-A/CSAG) subfamilies are coordinately upregulated in castrate-resistant PCa, but not in primary PCa. Interestingly, however, the CT-X antigen prostate-associated gene 4 (PAGE4) was found to be highly upregulated in primary PCa but silent in castrate-resistant PCa, thereby raising the possibility that CTA-based “gene signature” could potentially be developed to distinguish men with aggressive PCa who need treatment from men with indolent disease not requiring immediate intervention [48].

**Figure 1:**
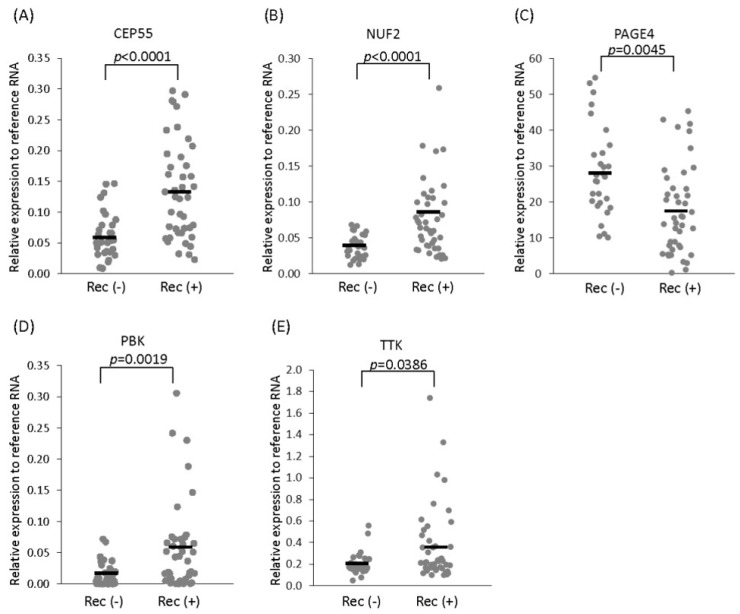
Cancer/Testis Antigen (CTA) expression in recurrent and non-recurrent prostate cancer. CTA expression in clinically localized prostate cancer with recurrence (Rec (+)) (*n* = 43) and without recurrence (Rec (−)) (*n* = 29). (**A**) centrosomal protein of 55 kDa (CEP55); (**B**) NDC80 kinetohore complex component NUF2; (**C**) prostate-associated gene 4 (PAGE4); (**D**) lymphokine-activated killer T-cell-originated protein kinase (PBK); (**E**) the dual specificity protein kinase TTK. Reproduced with permission from ref. [49].

**Figure 2:**
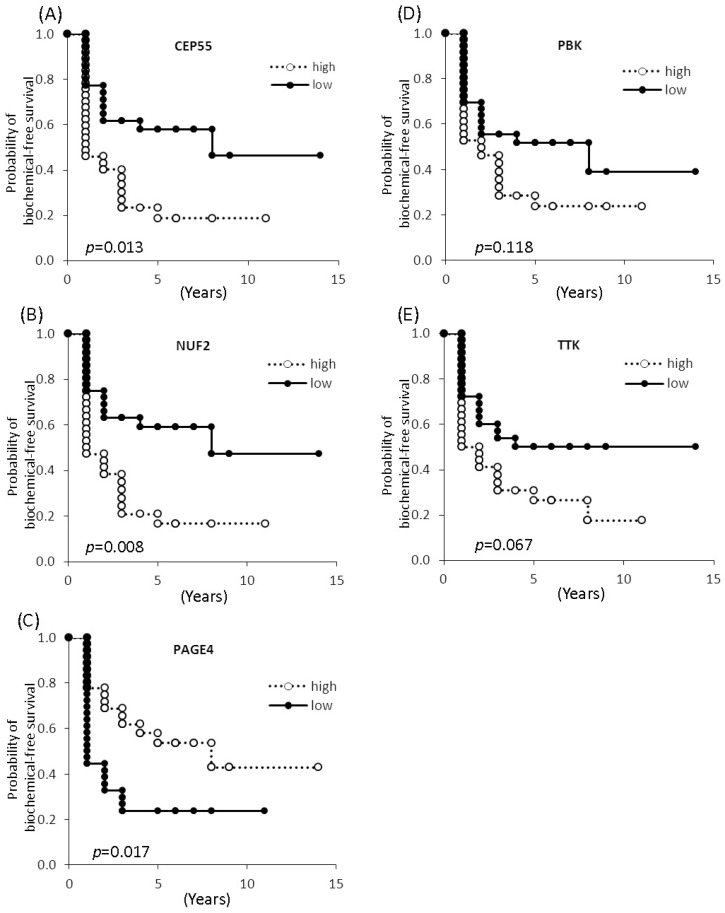
Kaplan-Meier analyses. Kaplan-Meier curves showing biochemical recurrence-free survival against time after radical prostatectomy stratified by the mRNA expression of (**A**) CEP55; (**B**) NUF2; (**C**) PAGE4; (**D**) PBK; and (**E**) TTK (high versus low groups dichotomized by median value). Reproduced with permission from ref. [49].

To test this possibility, Shiraishi et al. [49] devised a multiplex real-time polymerase chain reaction (PCR) assay. From a panel of 22 CTAs that showed differential expression, they selected a subpanel of 5 CTAs that included 4 non-X CT antigens (centrosomal protein of 55 kDa—CEP55, NUF2, lymphokine-activated killer T-cell-originated protein kinase—PBK and the dual specificity protein kinase TTK) and the CT-X antigen, PAGE4 [49]. The authors found that while the non-X CTAs were upregulated, the CT-X antigen, PAGE4, was downregulated in patients with recurrent PCa after radical prostatectomy (Figure 1). Kaplan-Meier curves revealed that higher levels of expression of CEP55 and NUF2 were significantly correlated with shorter biochemical recurrence-free time [49]. In contrast, higher expression of PAGE4 was significantly correlated with longer biochemical recurrence-free time (Figure 2). Further, with the exception of TTK, the other CTAs were significantly correlated with prostatectomy GS, but none were correlated with age, preoperative PSA and tumor stage [49]. It is important note that, like in the case of the genomics tests that include several cell cycle progression genes, the five CTAs used in the study by Shiraishi et al. [49] are also associated with the cell cycle and proliferation. In fact, some of the CTAs are common to both gene sets highlighting the potential of this CTA panel.

Despite the promise however, there are some limitations to the study by Shiraishi et al. [49]. First, the sample number was limited (*n* = 72), and they were not derived from patients who were consecutively and prospectively recruited for this study. Second, a high-risk cohort was used as a result of selection of specimens with large-volume tumors appropriate for frozen tissue collection, not reflecting contemporary, newly screened radical prostatectomy population. Third, there was no significant difference in the CT-X antigens (synovial sarcoma antigen X (SSX), synovial sarcoma X breakpoint 2 (SSX2), chondrosarcoma-associated gene 2/3 protein (CSAG2), melanoma-associated antigen 2 (MAGE-A2) and melanoma-associated antigen 12 (MAGE-A12)) between patients with or without recurrence [49]. However, von Boehmer et al. [50] observed that the CT-X antigen melanoma-associated antigen C2/Cancer-testis antigen 10 (MAGE-C2/CT10) may be a predictor of biochemical recurrence after radical prostatectomy, even though its expression was detected only in 3.3% of primary PCa samples.

More recently, the same research group employed the nCounter Gene Expression Assay (NanoString Technologies, Seattle, WA, USA) instead of quantitative multiplex PCR to evaluate the CTA gene signature in PCa patients [51]. The nCounter Analysis System utilizes a novel digital technology that is based on direct multiplexed measurement of gene expression and offers high levels of precision and sensitivity (<1 copy per cell). The technology uses molecular “barcodes” and single molecule imaging to detect and count hundreds of unique transcripts in a single reaction. Each color-coded barcode is attached to a single target-specific probe corresponding to a gene of interest. Mixed together with controls, they form a multiplexed CodeSet. The assay does not rely on enzymes for processing or amplification and enables highly sensitive detection and quantification of gene expression from a wide variety of sample types including direct measurement from purified total RNA, cell and tissue lysates, RNA extracted from FFPE samples and blood without globin mitigation.

The nCounter Analysis System is an integrated system comprised of a fully-automated assay and is designed to provide a sensitive, reproducible, quantitative and highly multiplexed method (up to 800 transcripts in one tube) with a wide dynamic range with superior gene expression quantification results when compared to real-time PCR without RNA purification, cDNA preparation, or amplification [51]. Of the 22 CTAs selected initially by Shiraishi et al. [49], Takahashi et al. [51] found that, in mRNA samples extracted from surgical samples, at least 5 CTAs (CEP55, NUF2, TTK, PBK, and PAGE4) appeared to be differentially expressed between metastatic and localized PCa both by quantitative PCR and the nanowire technology. As expected, CEP55 (*p* < 0.01), PBK (*p* < 0.01), NUF2 (*p* < 0.01) and sperm-associated antigen 4 (SPAG4) (*p* < 0.01) were significantly upregulated and PAGE4 (*p* < 0.01) was downregulated in metastatic PCa compared to localized disease. Further, using this assay FFPE samples, the authors found that RNA expression levels of the CTAs CSAG2 and nucleolar protein 4 (NOL4) were significantly higher in men with GS 8–10 disease than those with GS ≤ 4 + 3 disease [51]. By contrast, the RNA expression level of PAGE4 was lower in men with GS 8–10 disease than those with GS ≤ 6 disease. Notwithstanding the slight disparity in the CTAs that appear to discriminate disease progression, this study further demonstrated the potential of the CTAs as PCa biomarkers [51] using achieved samples.

## 3. A Vast Majority of CTAs Are Predicted to Be Intrinsically Disordered

**Figure 3:**
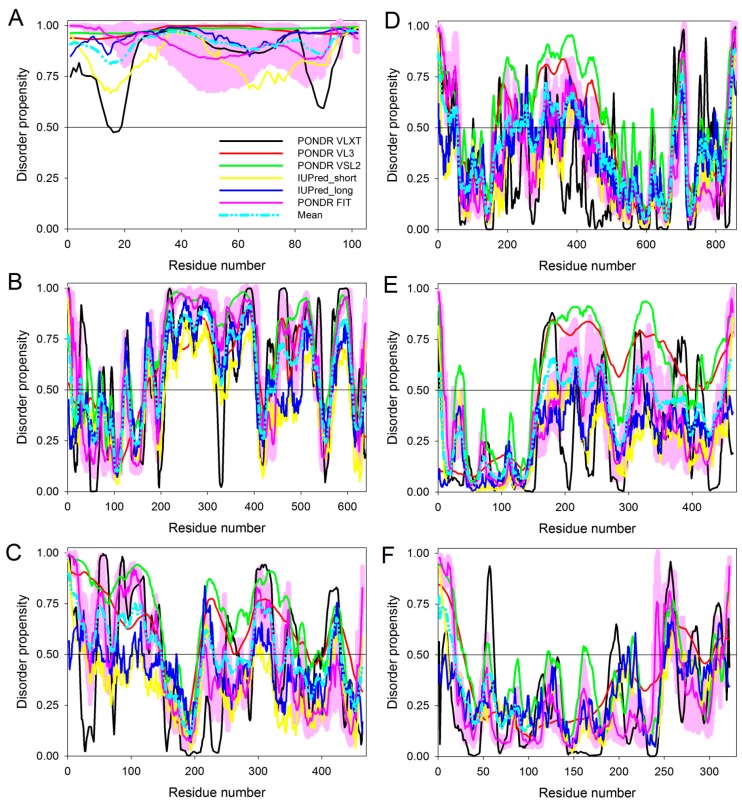
Variability of predicted intrinsic disorder levels and peculiarities of intrinsic disorder distributions within amino acid sequences of several CATs, PAGE4 (**A**, UniProt ID: O60829), nucleolar protein 4—NOL4 (**B**, UniProt ID: O94818), CEP55 (**C**, UniProt ID: Q53EZ4), TTK (**D**, UniProt ID: P33981), NUF2 (**E**, UniProt ID: Q9BZD4) and PBK (**F**, UniProt ID: Q96KB5). Intrinsic disorder profiles for query proteins generated by PONDR^®^ VLXT [113], PONDR^®^ VL3 [114], PONDR^®^ VSL2 [114,115], PONDR^®^ FIT [116], IUPred_short and IUPred_long [117] are shown by black, red, green, pink, yellow and blue lines, respectively. Cyan dash-dot-dotted lines show the mean disorder propensity calculated by averaging disorder profiles of individual predictors. Light pink shadow around the PONDR^®^ FIT curve shows error distribution. In these analyses, the predicted intrinsic disorder scores above 0.5 are considered to correspond to the disordered residues/regions, whereas regions with the disorder scores between 0.2 and 0.5 are considered flexible.

A bioinformatics study by Rajagopalan et al. [52] discovered that a majority of CTAs (>90%) are predicted to be intrinsically disordered proteins (IDPs). IDPs and hybrid proteins containing ordered domains and intrinsically disordered protein regions (IDPRs) are biologically active proteins that correspondingly lack rigid 3D structure either along their entire length or in localized regions, at least under physiological conditions in vitro [53,54,55]. Indeed, computational studies revealed that the per-proteome amounts of IDPs/IDPRs are high and increase with the increase in the organism complexity [56,57,58]. Indeed, all the CTAs selected by Shiraishi et al. [49] and Takahashi et al. [51] are predicted to be IDPs or as hybrid proteins containing long IDPRs (Figure 3 and Table 1). Despite the lack of unique structures, many IDPs/IDPRs can transition from disordered to ordered state upon binding to various targets [59]. The structural plasticity and conformational adaptability of IDPs/IDPRs, their ability to react and change easily and quickly in response to the changes in their environment, their capability to fold under the variety of conditions [53,54,55,60,61,62,63,64,65,66,67,68,69] combined with their binding promiscuity and unique capability to fold differently while interacting with different binding partners [66,70] define a wide set of functions exerted by IDPs/IDPRs in different biological systems. These same features determine the broad participation of IDPs/IDPRs in various biological processes [59,71,72] where they are involved in numerous signaling processes [73,74], regulation of different cellular pathways [75,76,77,78,79,80], cell protection [81], protein protection [82,83], cellular homeostasis [84,85] and cell cycle regulation [86,87,88,89,90]. Thus, biological activities of many IDPs/IDPRs are known to be precisely and tightly controlled and regulated by extensive posttranslational modifications (PTMs), such as phosphorylation, acetylation, glycosylation, etc. [59,91,92,93] and by alternative splicing (AS) [94,95,96].

Furthermore, IDPs/IDPRs are often associated with dosage sensitivity and are frequently engaged in highly promiscuous interactions, especially when concentrations of these proteins are increased [97]. Importantly, IDPs/IDPRs can interact with numerous binding partners of different natures, and many of these proteins are known to serve as essential hubs within various protein-protein interaction networks [68,98,99,100,101,102], where intrinsic disorder and related disorder-to-order transitions could enable one protein to interact with multiple partners (one-to-many signaling) or to enable multiple partners to bind to one protein (many-to-one signaling) [103].

Consistent with these observations, several CTAs are also predicted to bind DNA, and their forced expression appears to increase cell growth implying a potential dosage-sensitive function [52]. Furthermore, the CTAs appear to often occupy “hub” positions in protein-regulatory networks that typically adopt a “scale-free” power law distribution. Thus, the observations by Rajagopalan et al. [52] provide a novel perspective on the CTAs, implicating them in integrating and interpreting information in altered physiological states in a dosage-sensitive manner (see [52] and references therein). Considered together, these observations emphasizing the functional role of CTAs together with the development of a biomarker panel based on functionality (e.g., cell cycle progression), underscore the potential of CTAs to differentiate and discern diseased states of the prostate that is latent in their structure or lack thereof.

## 4. The Functional Role of Intrinsic Disorder in CTAs as Biomarkers

Here, using PAGE4 as an example, we illustrate how intrinsic disorder in CTAs may cast them as “next generation” biomarker candidates. More specifically, we discuss how conformational dynamics of this intrinsically disordered CTA may regulate the phenotype of the PCa cell and how the functionality of this IDP may be exploited as a potential biomarker in PCa.

PCa that is androgen-dependent is responsive to androgen-ablation therapy (ADT), the first line of treatment against advanced PCa, as well as an adjuvant to local treatment of high-risk disease. Although most patients initially respond to ADT, they eventually progress to a hormone-refractory state, which can prove fatal (reviewed in [104]). Yet the mechanism(s) underlying hormone resistance in PCa remains quite elusive. However, contrary to conventional wisdom, a new treatment paradigm called Bipolar ADT or BAT [105] is being tested where chemotherapy patients cycle through ADT followed by a supra-physiological dose of androgen. The results of this pilot study indicate that BAT may be more beneficial than ADT alone [105].

PAGE4 is a highly (~100%) intrinsically disordered CTA (Figure 3 and Table 1) that bears the hallmarks of a proto-oncogene; thus, it is highly expressed in the fetal human prostate, is undetectable in the normal adult gland, but is aberrantly expressed in androgen-dependent primary PCa and not in androgen-independent metastatic disease [106,107,108]. PAGE4 is also a strong potentiator of the transcription factor, AP-1, which is implicated in PCa [109] and is phosphorylated by two kinases namely, homeodomain-interacting protein kinase 1 (HIPK1) and CDC-like kinase 2 (CLK2). HIPK1 phosphorylates PAGE4 at predominantly T51, which is critical for its transcriptional activity [110]. In contrast, CLK2 is responsible for hyperphosphorylation of PAGE4 at multiple S/T residues. Furthermore, while HIPK1-phosphorylated PAGE4 potentiates AP-1, CLK2-phosphorylated PAGE4 attenuates its activity. Consistently, biophysical measurements indicate that HIPK1-phosphorylated PAGE4 exhibits a relatively compact conformational ensemble that binds AP-1 [111], while hyperphosphorylated PAGE4 is more expanded, resembles a random coil and is characterized by the diminished affinity for AP-1 [112].

AP-1 can negatively regulate AR activity [119,120], and AR inhibits CLK2 expression [112]. Furthermore, cells resistant to ADT often have enhanced AR activity (AR protein expression can increase >25 fold), suggesting a positive correlation between ADT resistance and AR activity [121]. Based on these interactions, Kulkarni et al. constructed a circuit representing the PAGE4/AP-1/AR/CLK2 interactions that drives non-genetic phenotypic heterogeneity in PCa cells and developed a mathematical model to represent the dynamics of this circuit [112]. The model predicts that this circuit can display sustained or damped oscillations; i.e., androgen dependence of a cell need not be a fixed state, but can vary temporally. Thus, the model suggests that an isogenic population of PCa cells displays a continuum of phenotypes with varying androgen-dependence. These cells can reversibly switch between androgen-dependent and androgen-independent states, without any specific genetic perturbation [112]. These findings appear to explain why BAT treatment appears more beneficial than ADT alone.

**Figure 4:**
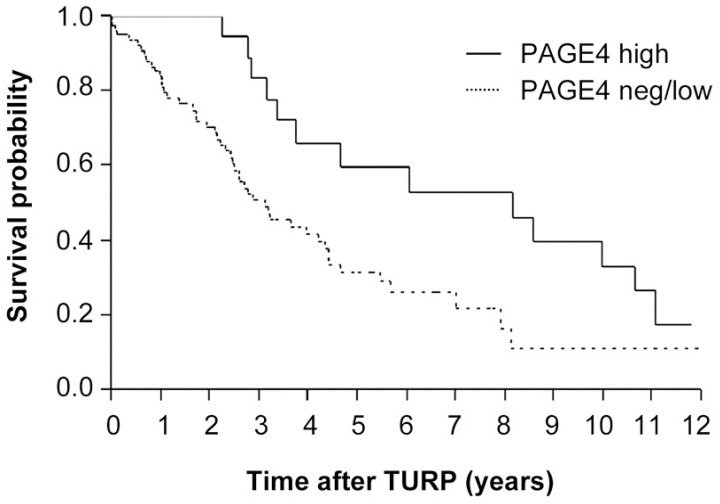
PAGE4 levels correlate with survival of patients with hormonenaive PCa. Overall survival of patients with hormone-naive PCa after transurethral resection of the prostate (TURP) for local advanced obstructive PCa stratified for high versus negative/low (neg/low) epithelial PAGE4 levels on the advanced PCa tissue microarray (TMA) (third quartile of mean epithelial PAGE4 intensity was set as the cut-off level). Reproduced with permission from ref. [107].

If this model is correct, then it suggests that higher levels of PAGE4 could be used as an indicator of a better PCa prognosis. Indeed, as noted by Shiraishi et al. [49], PAGE4 expression is significantly correlated with longer biochemical recurrence-free time (Figure 2). Furthermore, Sampson et al. [107] observed that in hormone-naive PCa, the median survival of patients with tumors expressing high PAGE4 levels was 8.2 years compared with 3.1 years for patients with tumors expressing negative/low levels of PAGE4 lending further credence to the model (Figure 4). Taken together, the work by Kulkarni et al. demonstrates a plausible functional link between IDP conformational dynamics and state switching in cancer [112]. Therefore, in theory, PAGE4 and its various phosphorylated variants represent novel biomarkers, as well as therapeutic targets to treat and manage PCa. For example, the detection of high levels of HIPK1-phosphorylated PAGE4 may imply that it can potentiate AP-1 and thus render the cells androgen sensitive and such patients may benefit from ADT alone. On the other hand, high levels of hyperphosphorylated PAGE4 would imply that the cells are heading towards an androgen-resistant state and thus, patients may benefit from BAT. Finally, pharmacologically targeting PAGE4 may also emerge as a viable option to treat PCa, especially low-risk disease.

**Figure 5:**
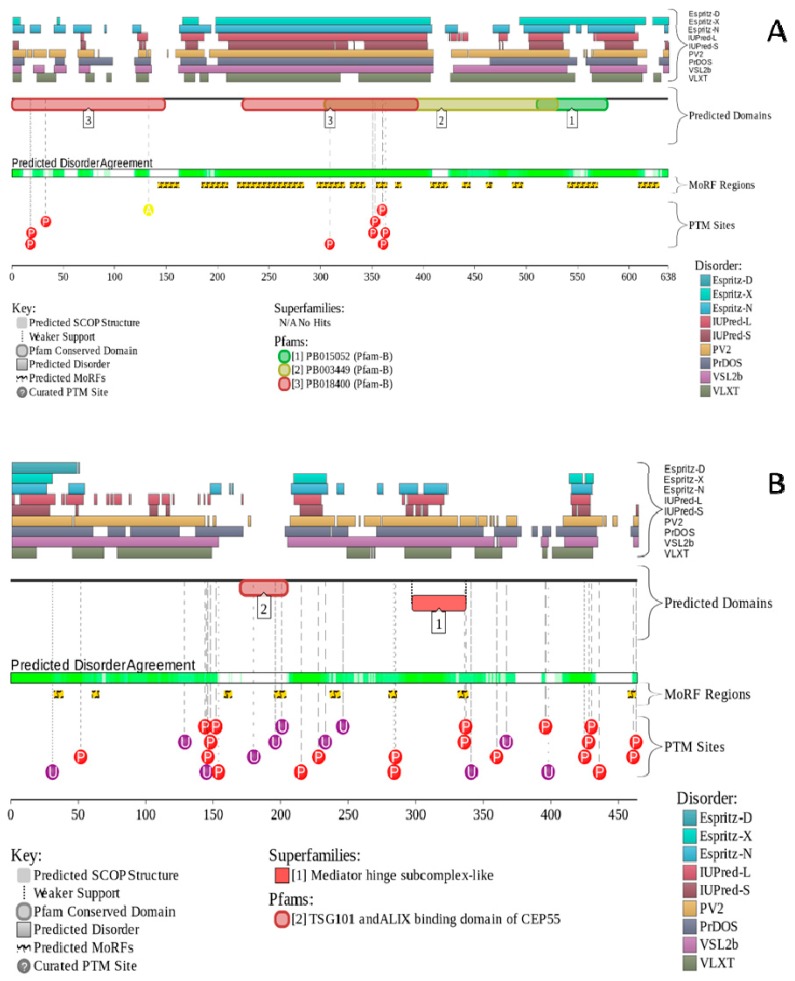
Intrinsic disorder propensity and some important disorder-related functional information generated for human NOL4 (**A**) and CEP55 (**B**) by the D^2^P^2^ database [127]. The D^2^P^2^ is a database of predicted disorder for a large library of proteins from completely sequenced genomes [127]. D^2^P^2^ database uses outputs of IUPred [117], PONDR^®^ VLXT [113], PrDOS [138], PONDR^®^ VSL2B [114,115], PV2 [127] and ESpritz [139] and is further supplemented by data concerning location of various curated posttranslational modifications and predicted disorder-based protein binding sites. Here, the green-and-white bar in the middle of the plot shows the predicted disorder agreement between nine predictors, with green parts corresponding to disordered regions by consensus. Yellow bar shows the location of the predicted disorder-based binding sites (molecular recognition features, MoRFs which are predicted by ANCHOR algorithm [140,141]), whereas colored circles at the bottom of the plot show location of various posttranslational modifications (PTMs).

Curiously, two other highly disordered CTAs, NOL4 and CEP55, were shown to be associated with different types of cancer. For example, aberrant methylation of CpG islands in the NOL4 gene promoter was shown to be associated with cervical [122] and head and neck squamous cell carcinoma (HNSCC) [123]. These studies showed that NOL4 is methylated in 85% of cervical cancers [122] and in 91% HNSCC samples [123] and therefore the analysis of the epigenetic alteration of this gene can be used for early detection and risk prediction of cancers. Furthermore, NOL4 was recently shown to be one of the 20 aberrantly expressed genes in the most common and the most lethal primary brain tumor, glioblastoma (GBM) [124]. Although the exact biological function of NOL4 protein is not known as of yet, recent analysis revealed that different AS variants of mouse NOL4 (canonical NOL4-L, NOL4-S that lacks the N-terminal tail of NOL4-L and NOL4-SΔ, a NOL4-S with missed nuclear localization signal (NLS)) differently regulate the transactivation activities of the transcription factors Mlr1 (Mblk-1-related protein-1, where Mblk stands for mushroom body large-type kenyon cell-specific protein) and Mlr2 [125]. According to UniProt [126], human NOL4 (UniProt ID: O94818) also might exist in 4 isoforms generated by AS, such as canonical form containing the full-length polypeptide chain, isoform-2 missing 413–514 region, isoform-3, where residues 1–87 (MESERDMYRQ...KQVLYVPVKT) are changed to a shorter sequence MADLMQETFLHHA and isoform-4 with missing N-terminal residues 1–285. Analysis of the functional disorder profile generated by the D^2^P^2^ platform [127] (see Figure 5A) suggests that the disorder-based functionality of this protein that includes the peculiarities of the PTM distribution and presence of the molecular recognition features (which are specific binding sites that undergo disorder-to-order transition at binding to biological partners) is dramatically affected by AS, providing further support to the important idea that functionality of NOL4 can be regulated by AS (see also Table 1).

Like NOL4, CEP55 is also known to be expressed in various cancers [128,129], being barely detectable in normal tissues except for testis and thymus [130]. In fact, enhanced levels of this protein can be found in breast carcinoma, colorectal carcinoma and lung carcinoma tissues [130], as well as in human gastric carcinoma [131], urinary bladder transitional cell carcinoma [132] and in lung and liver cancers [129]. This protein is also induced at all stages of cervical cancer [133]. Furthermore, in breast cancer, CEP55 is one of the 16 genes, genomic alterations of which may be involved in tumorigenesis and in the processes of invasion and progression of disease [134]. CEP55-derived peptides were shown to serve as suitable candidates for the vaccine therapy of colorectal carcinoma [135]. Aberrant expression levels of the CEP55 genes are known to serve as prognostic marker of the estrogen receptor (ER) positive breast cancer [136]. In HNSCC, genomic instability and malignant transformation might involve CEP55 activation by aberrantly upregulated Forkhead box protein M1 (FOXM1) [137]. In gastric cancer, CEP55 plays a role in the induction of cell transformation in the RAC-alpha serine/threonine-protein kinase (AKT) signaling pathway-dependent manner [131]. CEP55 regulates cytokinesis via interaction with the peptidyl-prolyl isomerase Pin1 followed by the Polo-like kinase 1 (Plk1)-mediated phosphorylation of CEP55 needed for the function of this protein during cytokinesis.

In fact, pathologic levels of Pin1 being associated with tumorigenesis [142] and with Plk1 activity being needed for the negative regulation of the CEP55 function in cytokinesis [143]. In the BRCA2-dependent manner, CEP55 forms CEP55-ALIX (ALG-2 interacting protein X, also known as programmed cell death 6 interacting protein) and CEP55-TSG101 (another component of the ESCRT-1 (endosomal sorting complex required for transport-1) complex) complexes during abscission, whereas cancer-associated mutations in BRCA2 disrupts these interactions leading to the enhanced cytokinetic defects [144].

CEP55 is known to homodimerize, likely via its coiled-coil domains that are also responsible for protein-protein interactions, and can directly interact with centrosome components [128]. In agreement with this hypothesis, and with the emphasized ability of CEP55 to be engaged in interaction with the ALIX (which is a protein associated with the ESCRT), structural analysis revealed that the 160–217 region of CEP55 forms a non-canonical coiled-coil dimer that binds the Pro-rich sequence of ALIX (residues 797–809) [145]. Although no structural information is available for the remaining parts of human CEP55, Figure 3D and Figure 5B and Table 1 show that this protein is predicted to contain high levels of intrinsic disorder. Furthermore, human CEP55 (UniProt ID: Q53EZ4) is expected to have two AS-generated isoforms [126], a canonical full-length form and an isoform-2 with the missing 401–464 region and the 389–400 region NQITQLESLKQL being changed to KNNTVGILETAS. Figure 5B and Table 1 show that alternative splicing causes elimination of several phosphorylation sites and one MoRF in human CEP55. In other words, it is likely that similar to NOL4, the physiological and pathological functionalities of human CEP55 can be modulated by AS.

## 5. Conclusions and Future Directions

Preliminary evidence in the literature indicates PAGE4 protein is detected in serum [146]. Although the authors evaluated PAGE4 as a biomarker to discern symptomatic and asymptomatic benign prostate hypertrophy (BPH), it is plausible that serum PAGE4 levels could discern PCa from normal and hence substitute for PSA given that, in the adult human male, PAGE4 is remarkably prostate-specific marker and is undetectable in the normal adult prostate [108,147]. Furthermore, it is even conceivable that a minimally invasive test could potentially also be developed to discern “good” (organ-confined/androgen-dependent disease) and “bad” (metastatic/androgen -independent disease) PCa given the positive correlation between PAGE4 and biochemical recurrence-free survival following radical prostatectomy. Additionally, monoclonal antibodies against the differentially phosphorylated forms of PAGE4 could be explored as novel tools to discern any correlation with disease prognosis. With advances in technology, estimating the levels of PAGE4 RNA and/or protein in CTCs using single-cell transcriptome (RNA-Seq) and single-cell westerns, respectively could be developed as minimally invasive tests for diagnosis and/or disease prognosis.

Although the corresponding data on the differential involvement of different AS isoforms of NOL4 and CEP55 in cancer are lacking at the moment, it is tempting to conjecture that in addition to intrinsic disorder and PTMs, structural and functional variability induced in proteins by AS represents an important feature that might have different roles in different cancers. In fact, AS was indicated as one of the cellular mechanisms (such as chromosomal translocations, altered expression, PTMs, aberrant proteolytic degradation and defective trafficking) that might cause pathogenic transformations in IDPs [148]. Furthermore, the indicated structural plasticity and multifunctionality of PAGE4, NOL4 and CEP55 are in line with the proteoform concept, according to which a functional protein product of a single gene exists in different molecular forms generated by genetic variations, alternative splicing and PTMs [149], as well as by intrinsic conformational plasticity and as a result of protein functioning [150].

Therefore, as opposed to current practice wherein any analyte such as a protein(s), RNA (structural, messenger, small interfering, long non-coding), DNA and its genetic and/or epigenetic modifications, metabolite(s) or circulating tumor cells themselves are selected as biomarkers merely based on their potential for disease diagnostics or prognostics, here we emphasize functionality as an additional trait in selecting a biomarker. For example, the standard biomarker for PCa is PSA, which is a kallikrein protease, whose function in the disease remains poorly understood. By contrast, PAGE4, which is a remarkably prostate-specific cancer/testis antigen in the adult male, is an IDP. Therefore, when overexpressed, PAGE4 can engage in promiscuous interactions resulting in pathological changes, that is, it is dosage-sensitive (see [52] and cross references therein). Furthermore, PAGE4 is a putative proto-oncogene that also appears to contribute to phenotypic heterogeneity in PCa cells due to its conformational plasticity. In other words, PAGE4 not only serves as a biomarker but also represents a therapeutic target (a theranostic). Therefore, PAGE4 and other examples of CTAs discussed here, by virtue of their functionality (for example, cell cycle progression), represent a set of “smart” biomarkers.

Considered together, these observations and considerations support an important notion: the analysis of the protein expression levels in biological fluids may not be the optimal focus of clinical proteomic research and that novel proteomic approaches are needed for the discovery of structure- and function-based next generation or smart biomarkers [151,152]. 

Since data presented in Figure 3 and Figure 5 and Table 1 are the results of computational analyses used to show that some CTAs (PAGE4, NOL4 and CEP55) could be IDPs, this raises a legitimate question of whether any current biological methods can be utilized to confirm that these putative IDPs are really intrinsically disordered in cancer cells. Although earlier on there was some skepticism about the existence of disorder in proteins in the crowded cellular environment, this has been refuted by several studies that demonstrate that IDPs remain disordered in vivo both in bacterial and mammalian cells using in-cell NMR [153,154,155]. Clearly, conducting detailed structural and functional characterization of CTAs in vitro and in vivo represents an important future direction in this field. Another important question is related to the existence of the PAGE4-AR (androgen receptor) axis, namely, are the expression levels of PAGE4 as a PCa biomarker associated with the AR expression in the tissue specimens collected from PCa patients? Unfortunately, currently there are no direct data correlating PAGE4 and AR levels. However, as indicated in [112], one might suspect that there is an inverse correlation between the two, since PAGE4 is downregulated in metastatic disease, whereas AR is known to be upregulated at protein and/or mRNA level. Obviously, finding an exact answer to this question constitutes a very important subject for future research. Finally, it would be important to know if there is a link between PAGE4 and resistance to anti-cancer drugs, such as abiraterone or enzalutamide. Although we are not aware of any publication addressing this issue, and do not have corresponding data, we suspect an inverse correlation, since PAGE4 is downregulated in androgen-independent PCa cells. Again, careful analysis of this subject should be conducted in the future. 

## Figures and Tables

**Table 1:** Intrinsic disorder-related characterization of some human Cancer/Testis Antigens that can be used as the prostate cancer biomarkers.

Protein	UniProt ID	Protein Length (N_AIBS_) ^a^	PONDR-FIT (%) ^b^	MobiDB Consensus (%) ^c^	Location (Length) of Long Disordered Regions ^d^	Location (Length) of AIBSs ^e^	N_int_^f^
PAGE4, P antigen family member 4	O60829	102 (4/64.7)	100.00	100.00	1–102	1–8 (8)	N.P.
						14–31 (18)	
						53–81 (29)	
						92–102 (11)	
NOL4, Nucleolar protein 4	O94818	638 (13/40.1)	65.98	51.72	201–261 (61) 273–295 (23) 343–403 (61) 503–535 (33) 576–603 (28)	142–162 (21)	9
						184–209 (26)	
						219–283 (65)	
						296–323 (28)	
						329–343 (15)	
						354–364 (11)	
						372–378 (7)	
						407–423 (17)	
						438–445 (8)	
						461–466 (6)	
						486–496 (11)	
						540–569 (30)	
						609–628 (20)	
CEP55, centrosomal protein of 55 kDa	Q53EZ4	464 (8/12.1)	42.24	26.29	1–26 (26)	32–38 (7)	102
						60–65 (6)	
						158–163 (6)	
						195–203 (9)	
						236–243 (8)	
						280–285 (6)	
						331–338 (8)	
						457–462 (6)	
TTK, Dual specificity protein kinase TTK	P33981	857 (8/7.7)	28.70	23.64	371–410 (40) 836–857 (22)	238–244(7)	72
						271–279 (9)	
						292–297 (6)	
						347–356 (10)	
						362–368 (7)	
						407–412 (6)	
						811–819 (9)	
						824–835 (12)	
NUF2, Kinetochore protein Nuf2	Q9BZD4	464 (1/3.0)	23.28	5.17	N.P.	278–291 (14)	48
PBK, Lymphokine-activated killer T-cell-originated protein kinase	Q96KB5	322 (3/8.7)	16.77	13.35	N.P.	268–275 (8)	30
						290–300 (11)	
						314–322 (9)	

^a^ N_AIBS_ (A/B) represents the number of potential disorder-based binding sites identified by the ANCHOR algorithm (AIBS, A) and the percentage of residues involved in disorder-based interactions (B). ^b^ Content of disordered residues (i.e., residues with the disorder propensity ≥0.5) in a protein based on the PONDR-FIT disorder prediction. ^c^ Content of predicted disordered residues in a protein based on the MobiDB consensus score. ^d^ Information on long disordered regions (i.e., disordered regions of at least 10 residues) was obtained based on the MobiDB consensus profile. ^e^ AIBSs are potential disorder-based binding sites identified by the ANCHOR algorithm. ^f^ N_int_, number of interactions as found using the BioGRID server [118]; N.P.: not present.
